# The Ability of the Yeast *Wickerhamomyces anomalus* to Hydrolyze Immunogenic Wheat Gliadin Proteins

**DOI:** 10.3390/foods11244105

**Published:** 2022-12-19

**Authors:** Paula Xiomara Méndez, José Antonio Uña, Soledad Vega-Fernández, María Ángeles Santos

**Affiliations:** 1 Facultad de Ciencias de la Salud, Universidad Autónoma de Santo Domingo, Santo Domingo 10105, Dominican Republic; 2 Departamento de Microbiología y Genética, Universidad de Salamanca, 37007 Salamanca, Spain

**Keywords:** yeast *Wickerhamomyces anomalus*, wheat gliadins, proteases hydrolyzing gliadins, immunogenic gliadins, gliadin immune disorders

## Abstract

Gliadins proteins make up around 30% of total wheat flour proteins. They are involved in many immune disorders affecting an increasing number of people who eat foods made with wheat flour. The triggering factor is the accumulation in the gut of immunogenic peptides derived from incomplete degradation of gliadins by gastric proteases. Previous research has revealed the effectiveness of sourdough-fermentation technology or related lactic acid bacteria in reducing wheat flour allergenic proteins. However, there are no single yeast cultures for producing reduced allergenicity wheat products. This study evaluated sourdough-related yeast *Wickerhamomyces anomalus* strains for their ability to hydrolyze gliadin proteins. All yeast strains were able to degrade gliadins and use them as carbon and nitrogen sources. The proliferation of the yeast strains depended on the gliadin addition; complete hydrolysis was observed after 24 h. The strain showing higher proteolytic activity fermented, acceptably wheat flour dough. The gliadin content of the leavened dough was reduced by 50%. Bread made from the *W. anomalus*-fermented dough showed a 78% reduction in immunogenic α-gliadins. 50% of the decrease was attributed to the proteolytic activity of the yeast cells, and the other 35% to the baking process. These results show the potential of the yeast *W. anomalus* as a starter for reducing immunogenicity wheat products.

## 1. Introduction

Wheat plays a special role in human nutrition. Through foods such as pasta, bread, and other bakery products, wheat is a source of carbohydrates, proteins, lipids, minerals, and vitamins in the daily diet [1]. Focusing on wheat proteins represent more than 20% of the total protein intake in the human diet [2]. According to the extraction process, they are classified into four groups known as the Osborne fractions: (i) albumins, soluble in water; (ii) globulins, soluble in saline; (iii) gliadins, soluble in an aqueous alcohol solution (60–70%); and (iv) glutenins, only soluble in alcohol if reducing agents are included [3,4].

Gliadins and glutenins are gluten proteins. Owing to the high content of proline (P) and glutamine (Q) amino acids, gliadins, glutenins, and the closely-related proteins in other cereals (secalins of rye, hordeins of barley, avenin of oats) are known by the generic name of prolamins [5]. Despite the close functional relationship of gluten proteins, gliadins and glutenins differ in their organization and structure.

Gliadins are monomeric proteins divided into four types based on their size and N-terminal sequence, ω5- (60–68 kDa), ω1-2- (32–35 kDa), α- and γ-gliadins (32–45 kDa). Conversely, glutenins are polymeric proteins linked through intra- and intermolecular disulfide bonds, ranking them among the largest proteins found in nature [5]. Glutenin polymers consist of high molecular weight glutenin subunits (HMW-GS) (60–68 kDa) and low molecular weight glutenin subunits (LMW-GS) (32–45 kDa) [3,4,5].

According to the structure, under hydration, glutenin polymers (HMW-GS as backbone and LMW-GS as branches) interact with gliadin monomers to build gluten polymers, determining the technological properties of flour dough, viscosity and the extensibility from gliadins and strength and elasticity from glutenins. Gluten proteins are also responsible for gas retention in fermentation processes, positively impacting the final product traits, volume, and quality [5,6].

However, foods made from wheat flour can negatively impact human health, triggering adverse reactions in genetically susceptible individuals. Major disorders associated with wheat flour-related food intake are celiac disease (CD), wheat allergy (WA), and non-celiac wheat/gluten sensitivity (NCWGS) [7,8,9]. The common factor that triggers these pathologies is the accumulation of peptides in the small intestine from partial digestion of wheat grain/fluor by gastric-intestinal proteases. The binding of peptides from gluten proteins (gliadins and glutenins) to T cells in individuals expressing the human leukocyte antigen DQ2 or DQ8 triggers DC [10]. Peptides from any wheat grain proteins (gliadins, glutenins, albumins, globulins) are incorporated by the dendritic cells of the intestine triggering the WA [8]. NCWGS is caused by peptides from gluten proteins, peptides from grain defense proteins (α-amylase/trypsin inhibitors), and other components (oligosaccharides and fructans) that activate innate immunity cells [11]. One of the first peptides recognized as the most resistant to proteolysis in the gastrointestinal tract is the well-known 33-mer peptide from the α-gliadins, considered the most immunogenic [12].

Different strategies have been explored to eliminate or reduce the content of immunogenic proteins in wheat flour-related foods, ranging from treatment with specific proteases, supplemental intake of probiotics (i.e., live bacteria or yeasts with proteolytic activity) to the development of wheat varieties or interspecific hybrids by specific breeding of lines carrying desired traits that produce grains with a lower content of immunogenic peptides [13,14,15,16].

In particular, sourdough-fermentation technology has given good results in reducing the content of immunogenic proteins in bread, other baked goods, and pasta [17,18,19,20]. The technology uses lactic acid bacteria (LAB) endowed with extracellular protease activity, isolated from natural sourdough [18]. Frequently, LAB used belongs to the genus *Lactobacillus* [21,22,23,24]. Several *Lactobacillus* spp. strains are inoculated into the dough at concentrations of ~1–5 × 10^8^ colonies forming unit (CFU) per g of dough [17,19,20]. Fermentation is performed for a long time, hours or even days, and after a short fermentation with baker’s yeast, the dough is baked [17,20,24]. To increase the hydrolyzing capacity of sourdough, LAB fungal proteases are also added [19,20,24]. To manufacture bread and pasta without gluten or reduced gluten content, ground LAB-fermented dough has been used [19,20]. The LAB proteolytic system consists of cell-wall-associated serine proteinases (endopeptidases), dipeptidases, and aminopeptidases [21,22]. The conversion of peptides to free amino acids and the subsequent amino acids catabolism is a central metabolic activity in LAB [25].

In addition to LAB, natural sourdough harbors both *Saccharomyces* and *non-Saccharomyces* yeasts, among which is the yeast *Wickerhamomyces anomalus* [26]. Although numerous studies have been performed on microbiological and biochemical characteristics of sourdough in the last three decades, few studies have been directed to know the proteolytic activity of sourdough yeasts [27].

**Figure 1:**
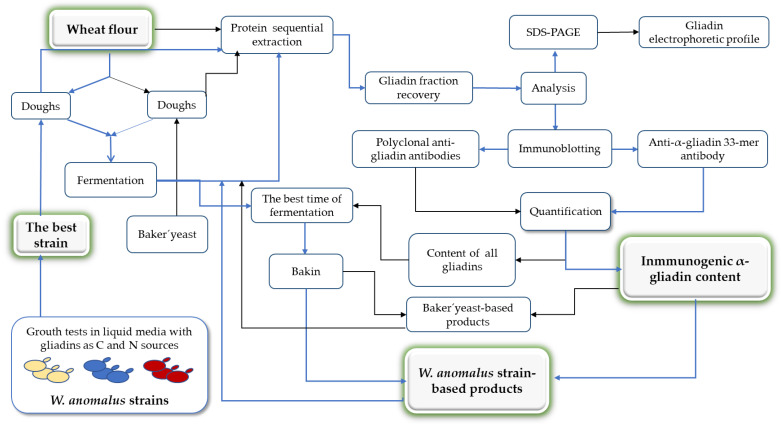
Workflow for exploring the ability of the yeast *W. anomalus* to hydrolyze immunogenic wheat gliadin proteins.

Recent reports provide data on the contribution of some yeasts to the degradation of immunogenic wheat proteins when co-inoculated with LAB [28,29]. However, no yeasts performed proteolysis alone have been reported to date. In this work, we explore the ability of the yeast *W. anomalus* itself to degrade gliadin proteins. In addition, we determine the yeast’s effectiveness in reducing immunogenic gliadin content in end-made products from wheat flour fermented with *W. anomalus* cells. The full workflow is shown as a flowchart (Figure 1).

## 2. Materials and Methods

### 2.1. Yeast Strains and Growth Conditions

*W. anomalus* yeast strains T9 and HRITi1 were isolated from tritordeum flour, ME1FP9 was isolated from wheat mother dough (hereinafter “ME1”), and YMAT1 was isolated from bakery dough, all of them from the PANLEV collection [30], were used in this work. Hércules baker’s yeast (*Saccharomyces cerevisiae*) by Lesaffre Ibérica (Valladolid, Spain) was used as the control.

The yeast strains were routinely grown in YPD (Yeast extract Peptone Dextrose) medium, 1% yeast extract, 2% Bacto Peptone, and 2% glucose. 2% agar was added to the solid medium. Liquid cultures were grown at 180 rpm in an orbital shaker. Inoculated media, solid and liquid, were incubated at 28 °C.

To test the ability of the yeasts to digest gliadin, the strains were cultured in a synthetic medium yeast nitrogen base without ammonium sulfate, and amino acids (SMW) (Formedium™, Ibian Technologies, Zaragoza, Spain) supplemented with 0.5% (*v*/*v*) wheat gliadins. In this medium, gliadins were the only source of nitrogen and carbon. Commercial pure gliadins (PGli) and gliadins extracted from commercial wheat gluten (GGli) were used. To prepare gliadin stock solutions, 10 g of gliadin or gluten power was added to 200 mL of 60% ethanol and incubated overnight at 22 °C on a shaker. After centrifugation for 5 min at 300× *g,* the supernatant containing the gliadin proteins was recovered and stored at 4 °C until use, as other authors have described [31]. Pure gliadins and gluten were purchased from Merck Life Science S.L.U., Sigma Co., Madrid, Spain. The standard synthetic medium, SM (Formedium™), with ammonium sulfate (5% *w*/*v*) and glucose (2% *w*/*v*) as the nitrogen and carbon sources, respectively, was used as control. The cultures were performed in microtiter plates (Nunc^TM^, Thermo Fisher, Madrid, Spain) (200 µL of medium/well) or Erlenmeyer flasks (100 mL of medium/500 mL Erlenmeyer flask). Respectively, 1 µL and 10 µL yeast cells from a preinoculum grown for 24 h in a YPD medium were used for inoculation. The evolution of the cultures was followed spectrophotometrically by periodically measuring the optical density at 600 nm (OD_600_) using the reader Multiskan GO microplate (Thermo Fisher, Madrid, Spain) or the Eppendorf BioSpectrometer^®^ (Eppendorf Ibérica S.L.U., Madrid, Spain). Three replicates per culture were performed. Growth is expressed as the OD_600_ mean of the replicates.

### 2.2. Wheat Flour Fermentation

A representative sample of bakery wheat flour, the V-25 flour from Harinera Vilafranquina (Barcelona, Spain), was selected. The flour was provided by Atrian Bakers S.L (Barcelona, Spain), and its physicochemical characteristics are 14.4% moisture, 12.1% protein, 368 s falling number, 27.2% wet gluten, 8.4% dry gluten, 90.5% gluten index (GI). In addition, tritordeum hybrid cereal flour, supplied by Atrian Bakers, and soybean flour (Merck Life Science S.L.U., Sigma Co., Madrid, Spain), were used as controls in some experiments.

V-25 flour dough was made by mixing 80 g of flour with 80 mL of sterile tap water (autoclaved) containing 1.6 g of NaCl. The dough was inoculated with yeast cells *W. anomalus* ME1 or Hércules baker’s yeast. Yeasts were omitted for the reference dough. For dough inoculations, the yeasts were grown in 50 mL of YPD medium for 24 h on the shaker, pelleted (3000× *g* for 10 min at room temperature), washed once with sterile deionized water, and resuspended in 10 mL of sterile tap water. Afterward, the concentration of the yeast cells (OD_600_) was determined in a spectrophotometer (Eppendorf BioSpectrometer^®^). Calibration OD_600_ to colony forming unit (CFU) counts resulted in 1 × 10^7^ *W. anomalus* CFU/mL and 1.3 × 10^7^ *S. cerevisiae* CFU/mL for 1 OD_600_. 5 × 10^7^ CFU per g of flour were used from both yeasts.

The fermentations were conducted for 24 h, or 48 h at 22 °C in sterile 250 mL graduated polypropylene or glass beakers covered with sterile gauze (4 caps) to avoid contamination and allow gas to escape. The rising of the dough was considered indicative of the fermentation activity. Samples of 0.1 g were taken periodically and kept in Eppendorf tubes at −20 °C until processing (Section 2.3).

### 2.3. Extraction of the Gliadin Proteins from Wheat Flour, Wheat Flour-fermented, and Bread

The sequential extraction procedure described by Schalk et al. [3] was applied. The sample (0.1 g) was weighed in an Eppendorf tube, and 1 mL of the salt solution (SS) (0.4 M NaCl, 0.067 M Na_2_HPO_4_/KH_2_PO_4_, pH 7.6) was added. The suspension was vigorously vortexed for 2 min, incubated at room temperature (RT) for 8 min, and then centrifuged (3750× *g*) for 20 min at RT. The supernatant was removed, and the sediment underwent a second round of extraction with 0.5 mL of SS. Both supernatants were combined, obtaining the albumin (soluble in water) and globulin (soluble in a salt solution) fraction (AG). Then, the fraction of gliadins was obtained from the AG-free sediment by extracting three folds with 0.5 mL of ethanol/water (60/40, *v*/*v*) for 10 min at RT, as described for the AG fraction. The extracted fractions were kept at −20 °C until analysis.

For analysis of the glutenin fraction, when needed, the last sediment from the step before was extracted threefold with 0.5 mL of the glutelin solution [2-propanol/water (50:50, *v*/*v*)/0.1 M Tris-HCl, pH 7.5, containing 2 M urea and 0.06 M dithiothreitol (DTT)], for 30 min at 60 °C (Dry Bath, Thermo Fisher, Madrid, Spain).

All chemicals and solvents were purchased from Merck Life Science S.L.U., Sigma Co. (Madrid, Spain).

Gliadin extraction from samples in the same experiment, i.e., flours and fermented flours (Section 3.4 assay) or fermented dough and bread (Section 3.5 assay), was done simultaneously to reduce the variability among samples.

Samples kept at −20 °C (Section 2.2 flour-fermented and Section 2.5 bread) were thawed at room temperature for 5 min before initiating the extraction of the gliadin proteins.

Protein concentration was determined by the standard Bradford assay (Bio-Rad Laboratories, Inc., Madrid, Spain) in 96-well plates (Nunc^TM^, Thermo Fisher, Madrid, Spain). The microtiter plates were read at 595 nm using the Multiskan GO microplate spectrophotometer (Thermo Fisher, Madrid, Spain).

### 2.4. One-Dimensional Sodium Dodecyl Sulfate-Polyacrylamide Gel Electrophoresis (SDS-PAGE) Analysis

To separate protein molecules based on size, the denaturing polyacrylamide gel system was used. The gels were prepared from a 30% acrylamide/bis-acrylamide (29:1) solution (Bio-Rad Laboratories, Inc., Madrid, Spain) using the devices of the Mini-Protean Tetra Cell vertical polyacrylamide gel electrophoresis system (0.75 mm) (Bio-Rad Laboratories, Inc., Madrid, Spain). An acrylamide concentration of 12% to running gel and 4.5% to stacking gel were used. The gel solutions and the tris-glycine electrophoresis buffer were prepared by standard methods [32].

The samples to be resolved were combined with the appropriate amount of Laemmli 4X loading buffer (VWR International Eurolab S.L., Barcelona, Spain), heated for 5 min in a 95 °C heat block to ensure complete protein denaturation, and were immediately loaded onto the gel, together with the prestained protein molecular weight marker sample (5 µL) (Spectra™ Multicolor Broad Range Protein Ladder, 10 to 260 kDa, Thermo Fisher, Madrid, Spain). SDS-PAGE was conducted with a constant current of 30 mA until the loading buffer dye ran off the gel (~1 h).

After electrophoresis, the gels were immersed in a solution of 15% methanol, 7.5% acetic acid (SMA), incubated with shaking for 5 min, stained with Coomassie^®^ Brilliant blue R 250 solution (SMA, 0.25% Coomassie, Merck Life Science S.L.U., Sigma Co., Madrid, Spain) for 10 min and washed twice with SMA until the protein bands became visible.

### 2.5. Bread Making

Dough preparation and fermentation were performed as indicated above. After fermentation, the dough was transferred with a spoon to individual stainless-steel flan molds (diameter 6.6 cm, height 4.5 cm) and baked in a home oven at 250 °C for 20 min. A moist atmosphere was provided to prevent the bread crust from excessively drying by putting a container full of water into the oven. Three small loaves of bread were made from each fermented dough.

Once the loafs cooled, they were sliced, and four small pieces (25 mg each) of the crumbs were combined to obtain representative samples of 0.1 g. All samples were kept in Eppendorf tubes at −20 °C until processing (Section 2.3).

### 2.6. Immunoblotting Analysis

Equal amounts (1 µL) of the gliadin fraction proteins were separated on SDS-PAGE and transferred into the Immobilon^®^-P membrane (Merck Life Science S.L.U., Sigma Co., Madrid, Spain) using the Mini Trans-Blot^®^ electrophoretic transfer (Bio-Rad, Madrid, Spain) and CAPS transfer buffer (20% methanol, 10 mM NaHCO_3_, 3 mM Na_2_CO_3_) at 100 V for 1 h.

The membranes were equilibrated for 5 min in 1X PBS (Bio-Rad, Madrid, Spain) containing 0.1% Tween^®^ 20 (*v*/*v*) (Merck Life Science S.L.U., Sigma Co., Madrid, Spain), blocked with 5% milk powder (*w*/*v*), 1X PBS, 0.1% Tween^®^ (MPBST solution) for 1 h at 22 °C and then incubated overnight at 4 °C with primary antibodies, the polyclonal anti-gliadin (wheat) antibody produced in rabbit (Merck Life Science S.L.U., Sigma Co., Madrid, Spain) diluted at 1/1500 in MPBST or the mouse monoclonal anti-gliadin antibody [14D5] (ab36729) (Abcam, Madrid, Spain) diluted at 1/1000. Subsequently, the membranes were washed three times with MPBST. They incubated 1 h at 22 °C with the secondary antibody (mouse anti-rabbit IgG-HRP or ProteinFind^®^ goat anti-mouse IgG (H+L)-HRP, both acquired from Quimigen (Madrid, Spain) also diluted in MPBST at 1/5000.

According to the product datasheet, the anti-gliadin antibody [14D5] (ab36729) reacts specifically with the synthetic peptide KLQPFPQPELPYPQPQ, which comprises the 33-mer sequence part of the wheat α-gliadin (residues 56–88).

Gliadin proteins that reacted with the primary antibody were identified by the chemiluminescence reaction of horseradish peroxidase (HRP) conjugated to the secondary antibody. The Clarity Western ECL Kit (Bio-Rad, Madrid, Spain) was used according to the manufacturer’s specifications. Proteins bands were visualized and analyzed using the ChemiDoc MP Imaging System and the Image Lab Software (Version 6.1) (Bio-Rad, Madrid, Spain).

The gliadin hydrolysis percentage for each sample was determined from the sum of all band intensities detected on the blot using the Image Lab Software. The formula [(Vi − Vt)/Vi] × 100 was applied, where “Vi” is the initial intensity value of the fermented samples at time 0 and “Vt” is the value of the fermented samples at 24 or 48 h. To determine the hydrolysis percentage in bread making, the same formula was applied using the Vi value of each dough for both fermented dough and bread samples. Vi and Vt are the average value of three independent experiments.

### 2.7. Statistical Analysis of Data

Yeast liquid cultures, wheat flour fermentation, and bread making, were performed in triplicate independent experiments. Data were captured on an Excel spreadsheet (Microsoft^®^ Excel^®^ for Microsoft 365 MSO, Version 2211). Mean ± standard deviation was calculated. Analysis of variance was applied to determine significant variations between means.

## 3. Results

### 3.1. Ability of W. anomalus Strains to Use Wheat Gliadins as Carbon and Nitrogen Sources

Previous work has revealed that *W. anomalus* strains can hydrolyze gliadins when they grow on a solid SM medium (Figure S1) [30]. However, it was unknown whether *W. anomalus* strains could use gliadins to supply their carbon and nitrogen needs.

**Figure 2:**
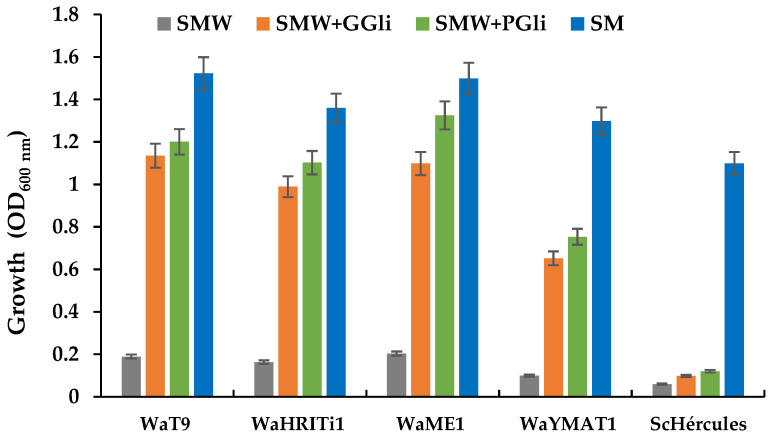
Growth of the *Wickerhamomyces anomalus* yeast in media with gliadin as the carbon and nitrogen sources. Representative *W. anomalus* strains WaT9, WaHRITi1, WaME1, and WaYMat1 were cultivated in microtiter plates for 4 days in the synthetic media without carbon and nitrogen sources (SMW, grey), SMW with wheat gliadins from gluten (SMW + GGli, orange) or pure wheat gliadins (SMW + PGli, green), and with carbon (glucose) and nitrogen (ammonium sulfate) sources (SM, blue). Baker’s yeast (ScHércules) was also cultivated in the same media. The bars represent the average OD_600_ ± standard deviation of three independent experiments.

Cultivation in microtiter plates of the *W. anomalus* strains T9, HRITi1, ME1, and MAT1 in the SMW medium without carbon and nitrogen sources supplemented with wheat gliadins (Section 2.1) revealed that strains can use these kinds of proteins to obtain the nitrogen and carbon necessary for their proliferation (Figure 2). Growth was similar with both wheat commercial wheat gliadins, pure gliadin fraction (PGli), and the gliadin fraction extracted from gluten (GGli). However, slight growth differences between both PGli and GGli media were observed. In contrast, consistent with the absence of extracellular hydrolytic activities as was seen in another study [30], the Hércules baker’s yeast *S. cerevisiae* could not grow in the media containing gliadins.

### 3.2. The Best W. anomalus Strain to Hydrolyze Wheat Gliadins

The T9 and ME1 strains which grew best with the two types of gliadins, were subjected to a new growth test. In this case, the culture was performed in Erlenmeyer flasks to better analyze the proliferative capacity of the strains in the culture media SMW + PGli and SMW + GGli.

**Figure 3:**
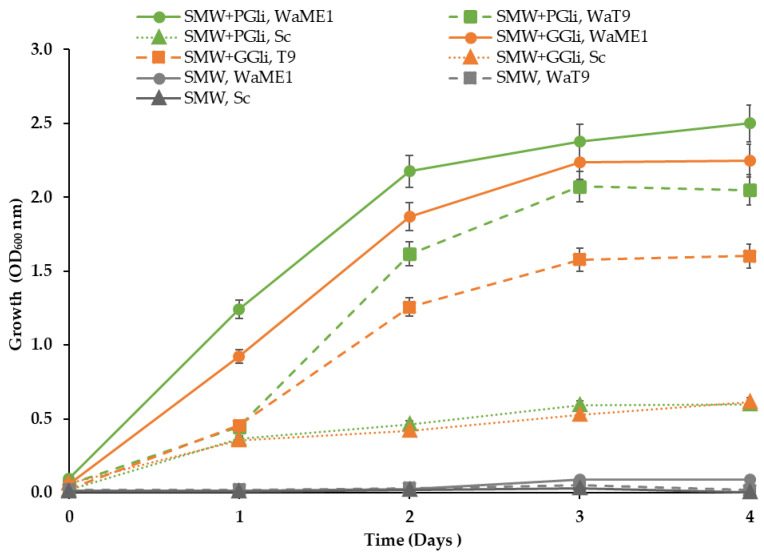
Ability to use gliadin proteins among *W. anomalus* strains. The growth of the *W. anomalus* strains ME1 (WaME1, solid line with circles) and T9 (WaT9, dashed line with squares) and the baker´s yeast (Sc, dotted line with triangles) in the media with gliadin proteins (SMW + PGli, green or SMW + GGli, orange) and without gliadins (SMW, grey) was monitored for 4 days. The points represent the average OD_600_ ± standard deviation of three independent experiments.

*W. anomalus* ME1 strain growth in the gliadin media was faster than the T9 strain (Figure 3). ME1 exhibited active growth immediately after inoculation, while the T9 strain did not actively grow until 24 h later. In addition, at 4 days, the ME1 strain cultures reached higher cell density than the T9 strain cultures, revealing that the ME1 strain has a greater capacity to hydrolyze gliadins. However, both strains showed differences between the cell density reached in the SMW + PGli medium (OD of the ME1 culture 2.5 and the T9 strain 1.6) and that obtained in the SMW + GGli medium (ME1 culture 2.2 and T9 culture 1.0).

**Figure 4:**
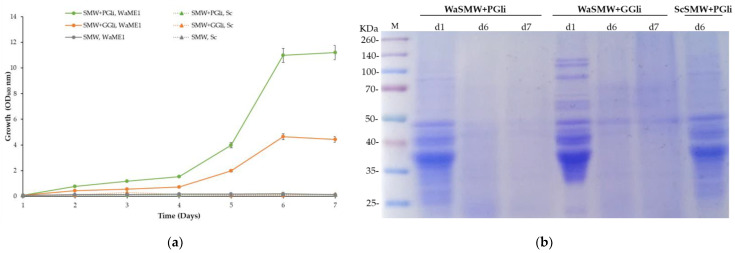
Time-course of *W. anomalus* ME1 strain growth by daily addition of gliadin proteins. (**a**) Growth of the *W. anomalus* ME1 strain (WaME1) and the baker’s yeast (Sc) in unfed (SMW) and fed (SMW + PGli or SMW + GGli) media. Synthetic media were fed daily with gliadins (0.5% *v*/*v*) from day 1 to day 6, both included. The points represent the average OD_600_ ± standard deviation daily of three independent experiments. (**b**) SDS-PAGE analysis of protein content in fed and unfed cultures. Samples were obtained by adding 10% trichloroacetic acid to 5 mL of culture after removing yeast cells by centrifugation. The protein pellet was resuspended in 25 µL of loading buffer, and half the sample (12.5 µL) was loaded onto the gel. The analysis of the samples taken from *W. anomalus* (Wa) cultures on days 1, 6, and 7 (d1, d6, d7) and from *S. cerevisiae* (Sc) cultures on day 6 (d6), just before adding the daily dose of gliadin proteins, is shown. M, protein mixture (25–260 kDa) was used as the molecular weight marker.

The difference between the ability to use the two types of gliadins, also observed in the experiment of the previous section (Figure 2), suggested a higher protein complexity in the fraction of gliadins extracted from gluten (GGli) than in the pure gliadins sample (PGli). Subsequent analyses of both gliadin types by SDS-PAGE confirmed the suggestion (Figure 4b).

### 3.3. The Ability of the W. anomalus Strain ME1 to Hydrolyze Wheat Gliadins Is Stable over Time

Cultures of the ME1 strain performed in SMW + PGli and SMW + GGli media showed continued active growth when fed daily with PGli and GGli, respectively (Section 2.1). Cell optical density (OD) doubled practically every day, with both cultures reaching the highest number of cells on day 6 (Figure 4a). From that day on, growth ceased, possibly due to the complete depletion of phosphorus, sulfur, or other trace elements in the SMW starting medium. Again, gluten-extracted gliadins (GGli) were found to be less efficient for growth (OD on day 6 = 4.6 ± 0.11) than pure gliadins (PGli) (OD on day 6 = 10.9 ± 0.11).

The SDS-PAGE analysis (Section 2.4) of protein samples, obtained by precipitation from cell-free culture, showed the ability of the *W. anomalus* strain ME1 to hydrolyze gliadins in both media, SMW + PGli and SMW + GGli (Figure 4b). Gliadin proteins were not observed in the ME1 strain samples on days 6 and 7. However, gliadins remained intact in samples taken immediately after adding *W. anomalus* (day 1). Gliadin proteins also remained unchanged in the *S. cerevisiae* strain Hércules culture samples, represented in Figure 4b by the sample taken on day 6.

Likewise, SDS-PAGE analysis revealed a different protein composition for the commercial gliadins used in the study (Section 2.1). PuGlia fraction is a less complex sample in which most proteins are distributed between the sizes of 25 and 50 kDa, as observed in the samples WaSMW + PGli on day 1 and ScSMW + PGli on day 6 (Figure 4b). While the GGli fraction contains considerable amounts of proteins over 50 kDa, and the proteins around 25 kDa are hardly represented (WaSMW + GGli sample on day 0). It is plausible that the different protein composition is the reason *W. anomalus* proliferates better in the medium with PGli since this fraction could be more easily hydrolyzed. PGli hydrolysis in the samples taken on days 6 and 7 seems to be complete, while in the GGli samples, some faint protein bands without degradation are observed.

The comparison between proliferation dynamics of *W. anomalus* in the unfed culture (Figure 3) with that exhibited in the fed culture (Figure 4a) and the SDS-PAGE analysis (Figure 4b) confirmed an efficient ability of the ME1 strain to use gliadins as a carbon and nitrogen sources. Furthermore, it became evident that the strain ME1 proteolytic activity to hydrolyze gliadin proteins is constant and stable over time.

### 3.4. The W. anomalus Strain ME1 Ability to Hydrolyze Gliadins in Wheat Flour Fermentation

After validating the reproducibility of the applied protocol to extract the gliadin fraction from wheat flour (Section 2.3 and Figure S2), we proceeded to analyze the effectiveness of *W. anomalus* in hydrolyzing gliadins during the fermentation process. To perform this task, dough made from bakery wheat flour (V-25) were inoculated with cells of *W. anomalus* (5 × 10^7^ CFU per g of flour) or with the same number of cells of the control yeast S. cerevisiae strain Hércules. They were incubated together with uninoculated dough at 22 °C (Section 2.2).

**Figure 6:**
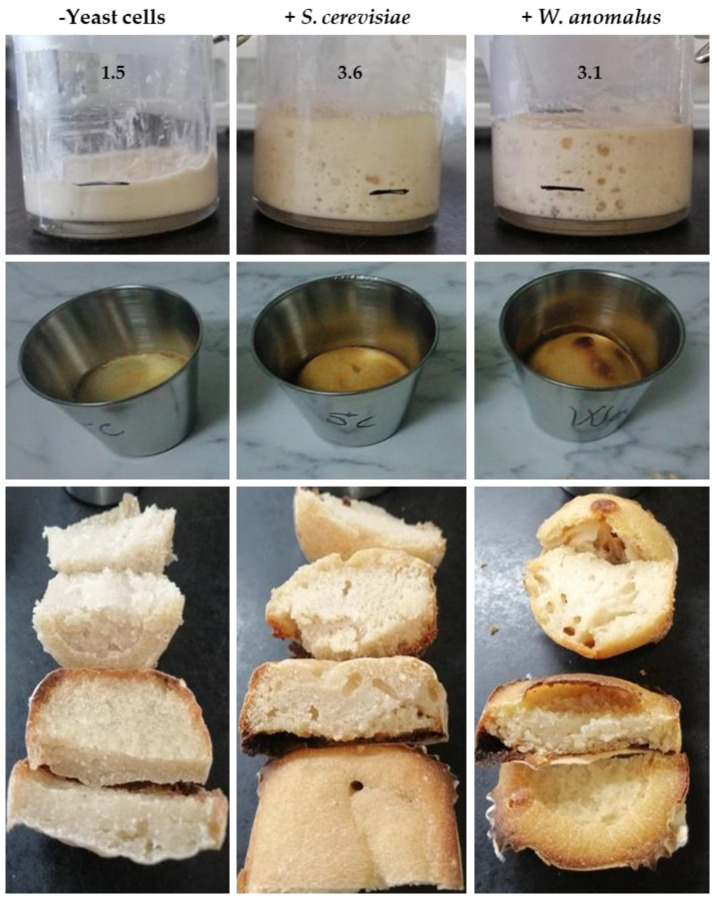
Wheat flour fermentation and bread making. In the top row, fermentation of the wheat flour dough uninoculated (− yeast cells) and inoculated (+) with the yeast *W. anomalus* ME1 strain or *S. cerevisiae* Hércules strain is shown. The number above the dough indicates the height reached, and the black line is the initial position of the dough. The left-hand image was taken at the end of fermentation (24 h) and the other two when the dough reached the maximum rise, the *W. anomalus*-inoculated dough at 14 h and the *S. cerevisiae*-inoculated at 10 h. The bread obtained from the fermented dough is shown in the photographs of the middle (after baking) and bottom (after slicing).

Periodic observation ensured that CO_2_ production, inferred from dough rising and bubbling, occurred only in yeast-inoculated dough and not in the uninoculated dough controls, in an equivalent way, as can be seen in Figure 6 (top row) of the next Section 3.5 experiment. The rise began at 8 h in *S. cerevisiae*-inoculated dough and reached maximum elevation at 10 h. However, in those inoculated with *W. anomalus*, the rise began later (at 12 h), reached maximum elevation later, at 14 h, and rose less (Figure 6, top row).

After fermentation, the gliadin fraction from equal sample amounts (0.1 g each dough), taken regularly, was extracted (Section 2.3), and the proteins obtained were resolved (10 µL) by SDS-PAGE (Section 2.4). Consistent with that already described, band analysis protocols of gliadins were simultaneously applied to unfermented wheat flour, tritordeum, and soybean flours (Section 2.2). These last two flours were used as controls to confirm that protein extraction from all types of samples (flours and dough) led to the specific recovery of gliadins.

**Figure 5:**
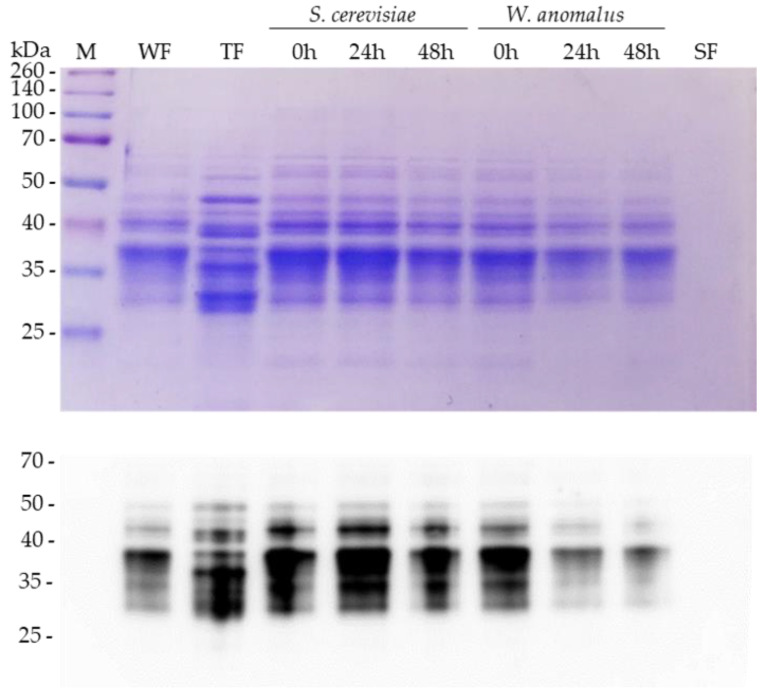
Hydrolysis of gliadins by *W. anomalus* in wheat flour fermentation. Top, SDS-PAGE analysis of the gliadin fraction extracted from unfermented flours (WF, TF, SF) and *S. cerevisiae*- or *W. anomalus*-fermented wheat flour (WF) dough. The fraction of gliadins extracted from samples taken at 0 h, 24 h, and 48 h after yeast inoculation is shown for fermented dough. WF, bakery flour V-25; TF, tritordeum hybrid cereal flour; and SF, soybean flour. Bottom analysis by immunoblotting of the gliadin fractions extracted from the duplicates of each sample analyzed at the top (flours and yeast-fermented dough). Gliadin proteins were detected by polyclonal anti-wheat gliadin antibodies.

A similar content of gliadins was recovered from the unfermented wheat flour (WF), from *S. cerevisiae*-dough samples (0 h, 24 h, 48 h), and the *W. anomalus*-dough 0 h sample (Figure 5, top image). However, the samples taken at 24 h and 48 h from *W. anomalus* dough revealed a lower content of gliadins, consistent with partial hydrolysis of the gliadin proteins. Consistent with that already described by other authors, tritordeum gliadins (TF) displayed a different protein pattern than wheat flour [16]. As expected, gliadin proteins were not obtained from soybean (SF) (Figure 5, last line) since soybean is a plant that cannot synthesize prolamin storage proteins [33].

The results were confirmed after extracting a new fraction of gliadins from each sample, separating the recovered proteins (SDS-PAGE), and transferring them to immobilon-P membrane for analysis by immunoblotting using polyclonal anti-wheat gliadin antibodies (Section 2.6). As can be seen in the Figure 5 immunoblot, the antibodies corroborated a lower content of gliadin proteins in the *W. anomalus*-fermented samples at 24 and 48 h. The hydrolysis percentage attributable to the yeast *W. anomalus*, quantified from the gliadins detected in the immunoblot (Section 2.6), was estimated at 50% (Table 1 and Table S1).

### 3.5. Incidence of the W. anomalus Hydrolytic Activity on the α-Gliadin Content in Wheat Bread

α-gliadins are the main proteins that elicit pathogenic immune responses and hypersensitivity reactions in susceptible people after gluten protein ingestion. Although several amino acid sequences may be involved in the adverse reactions, the most immunogenic region corresponds to the 33-mer named sequence harboring the α-gliadins [12].

In this regard, we wanted to know whether the *W. anomalus* ME1 strain´s hydrolysis ability could contribute to decreasing α-gliadin content in bakery products made from dough fermented with this yeast. To do this, first dough inoculated with the yeast *W. anomalus* or with the yeast *S. cerevisiae* were fermented for 24 h. As in the previous experiment (Section 3.4), bubbles and dough rising were only observed in those inoculated with any of the yeasts (Figure 6, top images).

Second, after fermentation, the dough was transferred to appropriate molds and baked (Section 2.5). No significant differences were detected between the bread loaves obtained from *W. anomalus*-fermented dough or those from *S. cerevisiae*-fermented dough. However, when the bread was sliced, the holes (gas cells) in the *W. anomalus* breadcrumbs were found to be fewer and larger than those in the *S. cerevisiae* breadcrumbs, possibly related to a lower content of gliadin proteins in *W. anomalus*-fermented dough (Figure 6, bottom images). As expected, the control bread from the uninoculated dough was smaller, with less volume and not gas cells (Figure 6, bottom images, left image).

Third, the gliadins from all dough and bread samples were simultaneously extracted (Section 2.2), then analyzed by SDS-PAGE. Finally, those gliadins that reacted with anti-α-gliadin 33-mer antibodies were detected.

**Figure 7:**
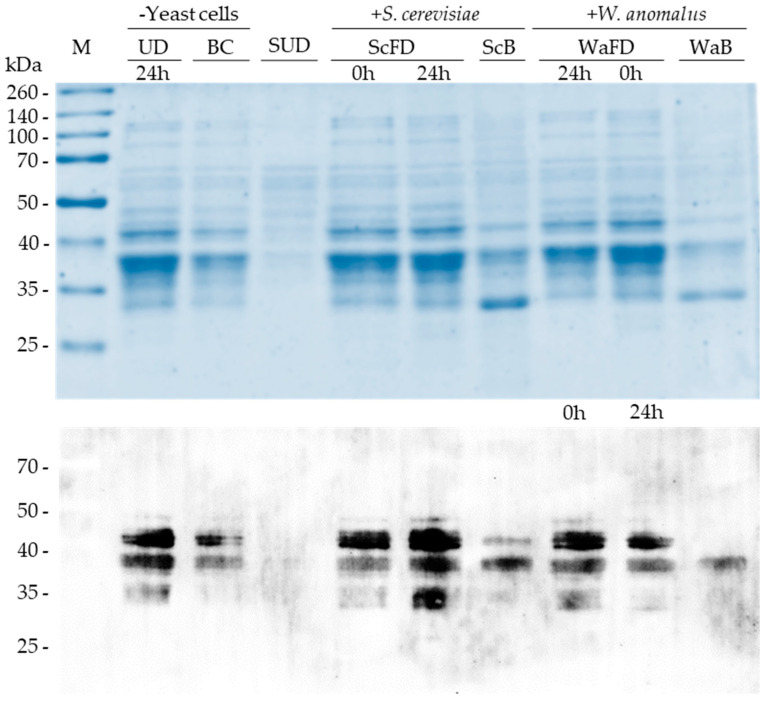
Detection of α-gliadin proteins in fermented dough and bread. The top image corresponds to the SDS-PAGE analysis of the gliadin fraction extracted from the dough and the bread shown previously (Figure 6). UD, unfermented dough; ScFD and WaFD, *S. cerevisiae*- and *W. anomalus*-fermented dough, respectively; BC, ScB, and WaB, bread obtained after dough UD, ScFD and WaFD were baked, respectively. The protein fraction of the soybean unfermented dough (SUD) was included as a negative control. The time at which the dough samples were taken is indicated. In the lower part, the immunodetection of the α-gliadins contained in each sample is shown. The monoclonal anti-α-gliadin antibody [14D5], which recognizes an amino acid sequence of the α-gliadin immunogenic peptide 33-mer, was used. Note that WaFD samples were loaded in a different order in the SDS-PAGE than the immunoblot.

The SDS-PAGE analyses revealed a lower content of gliadin proteins in all bread samples, bread produced from the control dough (BC) and bread obtained from the dough fermented with any of the yeasts (WaB or ScB) (Figure 7, top image). However, the protein pattern varied among bread samples. The control bread (CB) presented a gliadin pattern similar to that of unfermented dough (UD-24 h). On the contrary, the gliadin pattern of the ScB or WaB bread was different from that of the fermented dough from which they were obtained (ScFD 24 h, WaFD 24 h). Some protein bands were missing in bread samples (ScB and WaB), and new ones were seen, highlighting a new band smaller than the 35 kDa marker peptide in the ScB sample (see Figure 7, line ScB).

As in the previous SDS-PAGE analysis (Section 3.4), the samples from dough fermented for 24 h with *W. anomalus* (WaFD) showed a lower content of gliadins than the dough fermented with *S. cerevisiae* (ScFD).

The immunoblotting analyses showed that the antibody (Section 2.6) reacted with four proteins in the samples UD, BC, ScFD (0 h and 24 h), and WaFD (0 h and 24 h) (Figure 7, bottom image). Two of these proteins are slightly above the 40 kDa marker and migrate practically together, where there was no differentiation in some of the analyses. The other two proteins recognized are located below the 40 kDa marker and show a similar size, making it difficult to differentiate them in most samples.

However, the antibody recognized only three and two proteins in the ScB and WaB bread gliadin samples, respectively (Figure 7, bottom image). In ScB samples, the antibody did not detect the smaller of the two proteins above the 40 kDa marker. The detected protein was less represented in the sample than the other two proteins below the 40 kDa marker. In WaB samples, none of the proteins above the 40 kDa marker were detected, and the two proteins below 40 kDa were less represented than in the samples from the fermented dough (WaFD).

Possibly due to the high intensity of the bands, at first sight, no notable changes in the α-gliadin immunogenic peptide between the dough fermented with *W. anomalus* (WaFD 24 h) and unfermented dough (WaFD 0 h) were observed. However, quantification of the intensities (Section 2.6) revealed that the fermented sample (WaFD 24 h) contained 50% less immunogenic α-gliadins than the unfermented sample (WaFD 0 h) (Table 2 and Table S2). These results were consistent with those obtained in the previous experiment (Section 2, Table 1).

No antibody reaction was detected with proteins obtained from soybean flour (Figure 7, SUD sample), consistent with the expected specificity of the antibody to recognize the 33-mer epitope of wheat α-gliadin proteins (Section 2.6).

Other proteins (~35 kDa) in the dough samples UD, ScFD, and WaFD also reacted with the antibody, but the signal was weak compared to the intense and clear mark of the four proteins ~40 kDa, suggesting nonspecific reactions. Consequently, those proteins ~35 kDa would not harbor the 33-mer epitope of α-gliadins.

Considering the results of the SDS-PAGE and immunoblotting together, remarkable certainties can be extracted. (i) The dough-baking process decreases around 2.8-fold the content of the gliadin proteins in bread (Table 2). (ii) Dough fermentation for 24 h with *S. cerevisiae* Hércules strain or with *W. anomalus* MF1 strain modifies the pattern of gliadins in bread. (iii) *S. cerevisiae* fermentation activity has less incidence on the α-gliadin content in bread (56.7%) than yeast *W. anomalus* (78.1%) (Table 2 and Table S2). (iv) Among four α-gliadin proteins identified in the Spanish wheat flour V-25, *W. anomalus* ME1 strain fermentation activity promotes in bread the lack of 2 α-gliadins. It reduces the content of the other two.

## 4. Discussion

Among the yeasts isolated from sourdough worldwide, the yeast *W. anomalus* is one of the most common [34]. This yeast, previously known under the names *Hansenula anomala* or *Pichia anomala* [35], is also frequently isolated from flours and cereal grains [26,30]. These three natural habitats are well correlated with the ability of *W. anomalus* to use gliadin proteins as food.

The growth of *W. anomalus* from gliadin-media (SMW + PGli, SMW + GGli) is accompanied by protein hydrolysis. After one day, the hydrolysis of gliadins in the culture medium is practically complete, exhibiting intense proteolytic activity. The production of extracellular proteases by strains of the yeast *W. anomalus* has been repeatedly reported [36,37,38,39,40]. However, the ability to use these proteases to hydrolyze gliadin proteins and use the by-products for yeast growth itself has not been described before. To date, three extracellular proteases from *W. anomalus* have been identified [38,39,40]. Two are similar; they have a molecular size of around 30 kDa and maximum activity at pH 7–8, although they also have activity at lower pH [38,39]. The biochemical and structural data of the third protease indicate that it is an aspartyl protease with maximum activity at an acidic pH [40].

Although none of the *W. anomalus* extracellular proteases obtained have targeted gliadin proteins, it seems plausible that they participate in wheat gliadin hydrolysis when the yeast is cultivated in gliadin media. However, additional proteases may also be contributing. Indeed, neutral extracellular proteases with high hydrolytic activity targeting gluten proteins have been identified in *Bacillus subtilis* [41]. Extracellular aspartic proteases from filamentous fungi and yeasts have also been reported as effective proteolytic enzymes during the fermentation of several plant matrices [42].

According to the results of our study, a gliadin hydrolysis mechanism can be proposed for the *W. anomalus* ME1 strain. The yeast strain secretes moderate levels of proteases (endopeptidases and exopeptidases) in its growth environment. Protease production increases when insufficient levels of carbon or nitrogen are available and protein substrates are present, as in liquid gliadin media. The exopeptidases cleavage peptide bonds at the ends (N or C terminus) of gliadin polypeptides, liberating a single amino acid residue, a dipeptide, or a tripeptide. On the other hand, endopeptidases as aspartyl proteases cleavage peptide bonds in the inner regions. The coordinated action of both proteases would lead to complete gliadin polypeptide degradation.

After gliadin hydrolysis, *W. anomalus* cells must assimilate amino acids and degrade them to obtain carbon and nitrogen units from which they can synthesize macromolecules (proteins, carbohydrates, lipids, and so on). The ability of *W. anomalus* to use amino acids as carbon and nitrogen sources is shared with other members of the *non-Saccharomyces* yeast genera (*Candida*, *Debaryomyces*, *Pichia*, *Meyerozyma* or *Kluyveromyces*, among others) but not with yeasts from the *Saccharomyces* genus [43,44]. High activity of the NAD-dependent metabolic enzyme glutamate dehydrogenase 2 and methanol expression regulator appears to play a key role in the ability of *non-Saccharomyces* yeasts to assimilate C and N from amino acids [43,44]. It has been suggested that yeasts efficiently utilizing a wide variety of non-sugar-based substrates for growth have developed the ability to use amino acids as carbon and nitrogen sources. Certainly, all the strains of *W. anomalus* used in the work present growth rates in the media with gliadins like those they present in the standard SD medium, although some differences have been seen among the strains used in the work. The most efficient *W. anomalus* yeast is the ME1 strain, which was originally isolated from a wheat sourdough [30].

The sequential extraction procedure applied for extraction gliadins has made it possible to evaluate the impact of the *W. anomalus* action on the dough- and bread-gliadin contents. Furthermore, immunoblotting analysis has provided a more precise quantification of the degree of hydrolysis of the sample. However, other authors have commented that the commercial availability of antibodies limits the methodology to target other gliadin proteins [45]. Polyclonal antigliadin antibodies (Merck Life Science S.L.U., Sigma Co., Madrid, Spain) do not allow the detection of ꙍ-gliadins. Nevertheless, the monoclonal antigliadin antibody (ab36729) (Abcam, Madrid, Spain) seems specific for detecting α-gliadins harboring the 33-mer peptide.

The modest degree of gliadin hydrolysis led by *W. anomalus* in fermentation contrasts with that obtained when the yeast multiplies in gliadin media. Despite the culture media, the yeast proteolytic activity remains constant and stable over time, even after growth ceases (Figure 4a). In fermentation, the hydrolysis of gliadins only becomes evident after 24 h. Long fermentations are also required so that the proteolytic activity of lactic acid bacteria is appreciable in the sourdough [17,24,46]. However, an increase in fermentation time does not correlate directly with a higher degree of hydrolysis, as seen with *W. anomalus* at 48 h. Some authors consider that in these cases, increased of proteolysis is not detectable because peptidolysis is taking place; that is, proteases go on to hydrolyze undetectable small peptides with few amino acid residues [47].

Consistent with our findings, other authors have also reported the ability of *W. anomalus* to degrade gliadins during wheat flour fermentation [29]. A similar hydrolysis profile of all gliadins was seen, but 48 h of fermentation was necessary to achieve a degradation degree of 20%, ~2-fold lower than that obtained with the yeast strain *W. anomalus* ME1 at 24 h. Unlike our work, Sakandar et al. (2019) [29] detected oligopeptides and amino acids from gliadin hydrolysis since they used RP-HPL. In the same position, the authors achieved almost total gliadin hydrolysis when the yeast *W. anomalus* QAUWA03 and the *Enterococcus mundtii* QAUSD01 were used to ferment wheat dough.

A synergistic effect on the hydrolysis of wheat proteins in dough fermentation with co-cultures of the lactic bacteria *Pediococcus acidilactici* and the yeasts *S. cerevisiae* and *Torulaspora delbrueckii* has also been reported. However, its effect on gliadins has not been determined [28]. However, unlike *W. anomalus*, none of these yeasts exert any hydrolytic activity in fermentation when they are inoculated alone.

In the last decade, it has been shown that *non*-*Saccharomyces* yeasts can be used as an alternative to the traditional baker´s yeast *S. cerevisiae* in dough fermentations to produce baked goods [48,49]. According to our results, the strain of *W. anomalus* ME1 could be one of these yeasts. The bread obtained from *W. anomalus*-fermented dough had a similar appearance to those obtained from baker´s yeast-fermented dough. However, the dough with *W. anomalus* never rose as much as those with baker´s yeast, probably due to their lower CO_2_ production capacity [50]. However, this difference does not affect the final appearance since the bread obtained from both yeasts had a comparable size and shape.

In contrast, the decrease in gliadins in the *W. anomalus* bread appears to influence the number and size of gas cells in the crumb by the role of gliadins in the gas retention [51]. A more pleasant flavor (rustic bread flavor) was assigned to the bread produced from the *W. anomalus*-fermented dough when the bread was tested by the authors of the work, although none of them is an expert in bread tasting. The improvement could be associated with the diversity of volatile compounds produced by yeast *W. anomalus* during fermentation, some identified in sourdough bread [52,53,54].

Surprisingly, according to our results, the baking process reduces the gluten content in the bread. Experiments reveal that, after baking, the gliadin quantity is lower whether the bread is obtained from unfermented or fermented dough. However, this effect has not been explicitly reported before, although Brzozowski´s works seem to infer some contribution from the baking process to proteolysis in the bread samples [55]. Also, Vaquero et al. (2018) [16] found a marked decrease in gliadins in wheat and tritordeum bread after baking, although they did not analyze gliadin content before. Therefore, it is impossible to know if the decrease in gliadins was due only to baking or fermentation.

Our work indicates that fermentation and baking exert a synergistic effect, jointly contributing to reducing the content of gliadins. The contribution is much more pronounced in *W. anomalus*-made bread than in baker´s yeast-made bread. Before baking, gliadins from *W. anomalus*-fermented dough are already reduced (~50%), while in the baker´s yeast-fermented dough, the gliadin contents remain like that of the unfermented dough (Table 2 and Table S2). How both factors could have a synergistic action is something that we will have to study. A plausible explanation would be the heat activation of flour-specific proteases to which the intracellular yeast proteases released by cell lysis at the beginning of baking would bind. Endogenous flour proteases are activated by acidification induced by lactic acid bacteria in sourdough fermentations [22]. It has also been reported that high protease activity is released from lysed *S. cerevisiae* cells [56]. Our results show that baking contributes ~35% to gliadin degradation (Table 2 and Table S2).

The *W. anomalus* ME1 proteolytic activity helps to eliminate from bread two of four gliadins harboring the 33-mer peptide, which is considered the most toxic due to its resistance to human gastric proteases [12]. Furthermore, it makes it possible to reduce the content of the other two gliadins (Figure 7, bottom image). Among the diversity of gliadins analyzed from different wheat varieties, only two α-gliadins seem to harbor the 33-mer peptide [57,58]. However, four proteins similar two by two in wheat flour V-25 by the anti-33-mer antibody are recognized, consistent with two 33-mer-α-gliadin genes, each with two allelic versions. Amplification techniques and sequencing of the coding genes must verify this hypothesis.

As seen in this study, *W. anomalus* contributes to degrading the most immunogenic proteins in firm dough through fermentation. Still, its contribution may be even greater in liquid dough, given the hydrolysis degree observed in liquid gliadin media. On an industrial scale, liquid sourdough enables the obtaining of end-products with distinctive properties in taste and texture [59,60,61]. The incorporation of yeast *W. anomalus* into this technology could also contribute to reducing or eliminating gliadin content. In addition, the *W. anomalus* proteolytic activity could contribute to hydrolyzing other wheat proteins involved in non-celiac wheat sensitivity, such as α-amylase and trypsin inhibitory proteins, which specific lactic acid bacteria and yeasts can hydrolyze them [23,28]. However, this potential is yet to be explored.

## 5. Conclusions

Derived from the intake of gliadin proteins, human health can suffer many immune disorders affecting an increasing number of people. The ability of the yeast *Wickerhamomyces anomalus* ME1 to degrade gliadins can make this yeast an efficient tool to reduce or mitigate the negative gliadin effects. Through fermentation, the ME1 yeast, either alone or in combination with lactic acid bacteria or baker´s yeast, could eliminate many immunogenic gliadins in bakery goods. Additionally, the *W. anomalus* ME1 extracellular proteases may be used to eliminate food-contaminating gliadins that are added in the form of gluten or flour to confer viscoelastic or thickening characteristics to processed foods. However, exploiting the ability of *W. anomalus* ME1 requires determining the reproducibility of the results obtained with other flours and confirming in vivo the loss of immunogenicity of the *W. anomalus* ME1-based bread.

## Figures and Tables

**Table 1:** Gliadin hydrolysis percentage in wheat flour fermentation with *S. cerevisiae* and *W. anomalus*.

Time	*S. cerevisiae*	*W. anomalus*
0 h	0.0 ± 0.0	0.0 ± 0.0
24 h	1.2 ± 1.0	50.6 ± 1.7
48 h	1.3 ± 0.4	49.6 ± 1.1

**Table 2:** α-Gliadin hydrolysis percentage in bread making with baker’s yeast and *W. anomalus*.

Sample	Control	*S. cerevisiae*	*W. anomalus*
Fermented dough	0.0 ± 0.0	4.0 ± 1.5	50.8 ± 4.8
Bread	35.2 ± 13.5	56.7 ± 4.1	78.1 ± 2.7

## Data Availability

The data sets generated during the current study are available from the corresponding author on reasonable request.

## Supplementary Materials

The following are available online at https://www.mdpi.com/article/10.3390/foods11244105/s1, Figure S1: *W. anomalus* ME1 strain´s ability to hydrolyze wheat gliadin proteins on solid medium, Figure S2: Validation of the method used to obtain gliadin proteins, Figure S3: Gluten proteins of the wheat flour V-25 [3], Table S1: Quantification of gliadin proteins in wheat flour fermentation, Table S2: Quantification of gliadin proteins in bread making.
