# Supplementary material for: The Ability of the Yeast Wickerhamomyces anomalus to Hydrolyze Immunogenic Wheat Gliadin Proteins
Source: Foods. 2022 Dec 19;11(24):4105. doi: 10.3390/foods11244105 (PMC9778486; doi:10.3390/foods11244105)
Supplement: Supplementary file 1 [file foods-11-04105-s001.zip › foods-2089300-supplementary.pdf]

foods

Supplementary materials

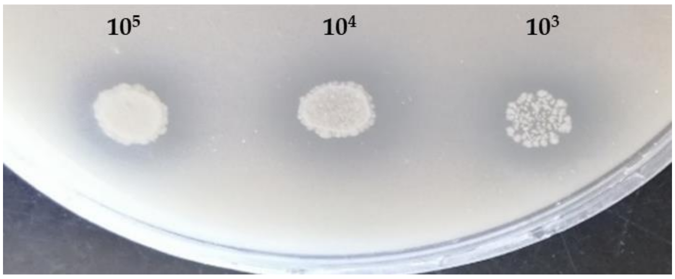

Figure S1. ***W. anomalus*** **ME1 strain´s ability to hydrolyze wheat gliadin proteins** on solid medium SM with glucose (2% w/v) and ammonium sulfate (5% w/v) as carbon and nitrogen sources, respectively, and gliadins (0.5% v/v).

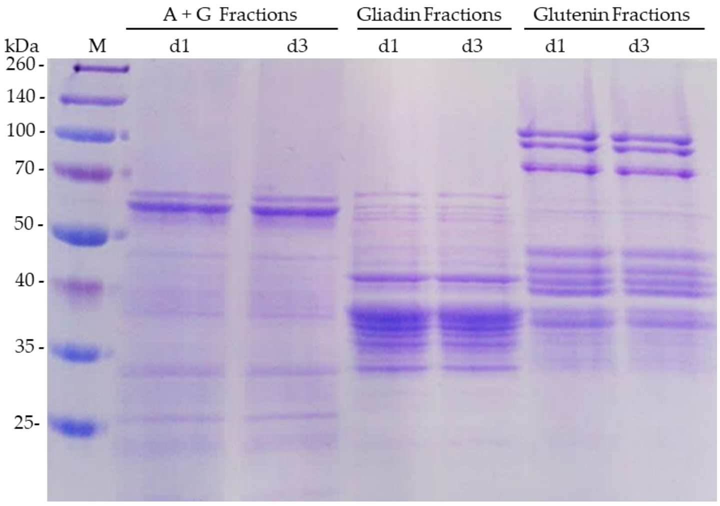

Figure S2. Validation of the method used to obtain gliadin proteins. The procedure described in Section 2.2 was applied to 3 samples of V-25 bakery wheat flour. One flour sample was processed per day. The protein fractions obtained on days 1 (d1) and 3 (d3) were resolved by SDS-PAGE (8 µL) and the protein profiles were compared. The gliadin protein fraction extracted with ethanol/water (60/40 v/v) was obtained after subtracting from the flour samples the albumin and globulin proteins (A+G fraction) with a salt solution. Glutenin proteins

extracted from the gliadin-free sediment with propanol-urea-DTT are also shown. M, protein mixture (25-260 kDa) used as the molecular weight marker.

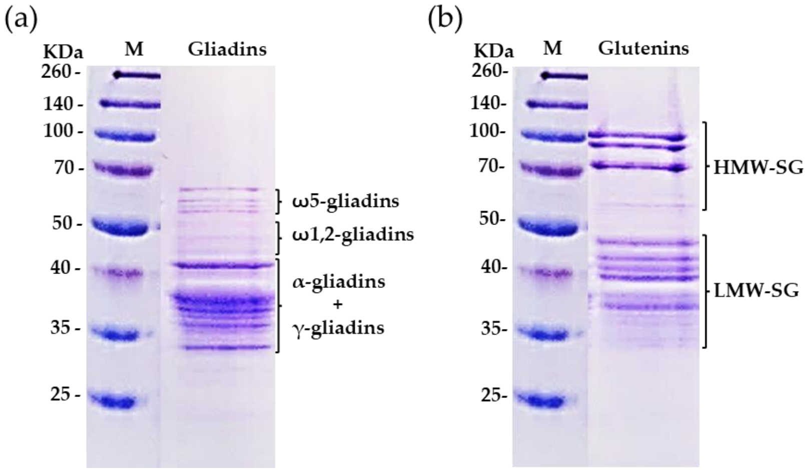

Figure S3. Gluten proteins of V-25 wheat flour. The bands belonging to the different types of gliadin (**a**) and glutenin (**b**) proteins were inferred from electrophoretic mobility pattern and molecular weights (M_r), according to those described for wheat flour samples in other works [3]. M_r ranges: ω5-gliadins 60 -68 kDa; ω1,2-gliadins 43-60 kDa; α- and γ-gliadins 32-45 Da; HMW-GS (high-molecular-weight-glutenin subunits) 80-120 kDa; LMW-GS (low-molecular-weight-glutenin subunits) 32-45 kDa. M, protein mixture used as M_r marker.

Table S1. Quantification of gliadin proteins in wheat flour fermentation from the bands identified by polyclonal antigliadin antibodies.

Sample	Fermentation time (hours)	Intensity of all bands (arbitrary units) per experiment (Exp)			Gliadin hydrolysis percentage per experiment (Exp)			Hydrolysis percentage average	Standard deviation (±)
		Exp 1	Exp 2	Exp 3	Exp 1	Exp 2	Exp 3		
Sc-fermented dough	0	39354795.0	37254873.0	38456213.0	0.0	0.0	0.0	0.0	0.0
Sc-fermented dough	24	39651672.0	38131462.0	38651321.0	0.8	2.4	0.5	1.2	1.0
Sc-fermented dough	48	38873034.0	37913032.0	38073045.0	1.0	1.8	1.0	1.3	0.4
Wa-fermented dough	0	38959146.0	38052346.0	38501571.0	0.0	0.0	0.0	0.0	0.0
Wa-fermented dough	24	19748473.0	19026173,0	18250785.5	49.3	50.0	52.6	50.6	1.7
Wa-fermented dough	48	19535562.0	19615581.0	19050643.0	49.9	48.5	50.5	49.6	1.1

Sc, Hércules baker's yeast; Wa, the *Wickerhamomyces anomalus* yeast strain ME1

Table S2. Quantification of gliadin proteins in bread making from the bands identified by the monoclonal anti-α-gliadin antibody [14D5] (ab367299).

Sample	Fermentation time (hours)	Intensity of all bands (arbitrary units) per experiment (Exp)			Gliadin hydrolysis percentage per experiment (Exp)			Hydrolysis percentage average	Standard deviation (±)
		Exp 1	Exp 2	Exp 3	Exp 1	Exp 2	Exp 3		
Uninoculated dough	24	2593188.0	2194236.0	1994760.0	0.0	0.0	0.0	0.0	0.0
Bread control		1366794.0	1739556.0	1242540.0	37.7	20.7	47.3	35.2	13.5
Sc-fermented dough	0	2776971.2	2598633.6	2547680.0	0.0	0.0	0.0	0.0	0.0
Sc-fermented dough	24	2710476.4	2710476.4	2683640.0	5.3	4.3	2.4	4.0	1.5
Sc-made bread		1275716.0	1177584.0	981320.0	61.4	54.7	54.1	56.7	4.1
Wa-fermented dough	0	2655180.0	2413800.0	2413800,0	0.0	0.0	0.0	0.0	0.0
Wa-fermented dough	24	1368640.0	1263360.0	1052800.0	56.3	47.7	48.5	50.8	4.8
Wa-made bread		640360.0	548880.0	457400.0	81.0	77.23	75.8	78.1	2.7

Sc, Hércules baker's yeast; Wa, the *Wickerhamomyces anomalus* yeast strain ME1
